# Supplementary material for: Pseudoislet Aggregation of Pancreatic β-Cells Improves Glucose Stimulated Insulin Secretion by Altering Glucose Metabolism and Increasing ATP Production
Source: Cells. 2022 Jul 29;11(15):2330. doi: 10.3390/cells11152330 (PMC9367366; doi:10.3390/cells11152330)
Supplement: Supplementary file 1 [file cells-11-02330-s001.zip › cells-1812680-supplementary.pdf]

Table S1: Donor Information for human islet preparations

Identifier	Age	Gender	BMI	Diabetes	HbA1c	Donor type	Centre
LDIS244	49	F	26.6	N	5	DCD	Newcastle
LDIS247	48	F	32.5	N	N/A	DBD	Kings
LDIS251	53	M	25	N	5.2	DBD	Newcastle

Table S2: Taqman sequences for real-time RT-PCR

Gene	Gene Symbol	Catalogue number
Glucose transporter protein type 1	Slc2a1	Mm00441480_m1
Pyruvate dehydrogenase kinase 1	Pdk1	Mm00554300_m1
Lactate dehydrogenase A	Ldha	Mm01612132_g1
Ribosomal protein lateral stalk subunit P0	RPLP0	Mm00725448_s1
